# Polymorphisms in the *ERCC5* Gene and Risk of Esophageal Squamous Cell Carcinoma (ESCC) in Eastern Chinese Populations

**DOI:** 10.1371/journal.pone.0041500

**Published:** 2012-07-26

**Authors:** Mei-Ling Zhu, Ting-Yan Shi, Hai-Chuan Hu, Jing He, Mengyun Wang, Li Jin, Ya-Jun Yang, Jiu-Cun Wang, Meng-Hong Sun, Huan Chen, Kuai-Le Zhao, Zhen Zhang, Hai-Quan Chen, Jia-Qing Xiang, Qing-Yi Wei

**Affiliations:** 1 Department of Oncology, Fudan University Shanghai Cancer Center, Shanghai, China; 2 Department of Oncology, Shanghai Medical College, Fudan University, Shanghai, China; 3 Cancer Research Laboratory, Fudan University Shanghai Cancer Center, Shanghai, China; 4 Department of Thoracic Surgery, Fudan University Shanghai Cancer Center, Shanghai, China; 5 State Key Laboratory of Genetic Engineering and MOE Key Laboratory of Contemporary Anthropology, School of Life Sciences and Institutes of Biomedical Sciences, Fudan University, Shanghai, China; 6 Department of Pathology, Fudan University Shanghai Cancer Center, Shanghai, China; 7 Department of Radiotherapy, Fudan University Shanghai Cancer Center, Shanghai, China; 8 Department of Epidemiology, The University of Texas MD Anderson Cancer Center, Houston, Texas, United States of America (JX); University of Nebraska Medical Center, United States of America

## Abstract

**Background:**

Excision repair cross complementing group 5 (*ERCC5 or XPG*) plays an important role in regulating DNA excision repair; its functional single nucleotide polymorphisms (SNPs) may alter DNA repair capacity and thus contribute to cancer risk.

**Methodology/Principal Findings:**

In a hospital-based case-control study of 1115 esophageal squamous cell carcinoma (ESCC) cases and 1117 cancer-free controls, we genotyped three potentially functional SNPs of *ERCC5* (SNPs, rs2296147T>C, rs2094258C>T and rs873601G>A) and estimated crude and adjusted odds ratios (ORs) and 95% confidence intervals (CIs) for their associations with risk of ESCC using unconditional logistic regression models. We also calculated false-positive report probabilities (FPRPs) for significant findings. We found that compared with the TT genotype, *ERCC5* rs2296147 C variant genotypes were associated with a significantly lower ESCC risk (CT: adjusted OR = 0.76, 95% CI = 0.63–0.93, CT/CC: adjusted OR = 0.80, 95% CI = 0.67–0.96); however, this risk was not observed for the other two SNPs (rs2094258C>T and rs873601 G>A), nor in further stratification and haplotype analysis.

**Conclusions/Significances:**

These findings suggested that *ERCC5* polymorphisms may contribute to risk of ESCC in Eastern Chinese populations, but the effect was weak and needs further validation by larger population-based case-control studies**.**

## Introduction

Esophageal cancer is the sixth leading cause of cancer-related death worldwide. In 2008, there were 482,300 new cases, and 406,800 patients died of this disease around the world. In the highest incidence areas that extend from northern Iran and through the central Asian republics to north-central China, esophageal squamous cell carcinomas (ESCC) account for 90% of the cases [1]. Although cigarette smoking, alcohol intake, nutritional deficiencies and dietary carcinogen exposure may contribute to the etiology of ESCC [2], only a small fraction of exposed individuals develop ESCC, suggesting that genetic susceptibility may also play a vital role in the etiology of ESCC [3], [4]. Recently, genetic polymorphisms in genes involved in multiple biological pathways, including DNA repair, have been identified as potential risk factors for ESCC [5], [6].

DNA repair is essential for maintaining genomic stability in response to the insults of environmental carcinogens that causes DNA damage; if left unrepaired, such DNA damage can lead to mutation fixation and initiation of carcinogenesis. To date, there are at least five known major DNA repair pathways, including more than 150 human DNA repair genes, among which the nucleotide-excision repair (NER) pathway is the most versatile and particularly important in association with cancer risk. This is because NER repairs several kinds of bulky and helix-distorting lesions in DNA, involving coordinated actions of damage detection and recognition by helicase and nuclease proteins [7]. More importantly, NER defects can lead to severe diseases, such as xeroderma pigmentosum, Cockayne syndrome and tri-chothiodystrophy, and some of these patients are prone to cancer [8], [9].

The excision repair cross complementing group 5 (*ERCC5*) gene, also known as the xeroderma pigmentosum group G (*XPG*) gene, is an indispensable component of NER, which belongs to the flap structure-specific endonuclease 1 (FEN1) family. *ERCC5* encodes a single-strand specific DNA endonuclease that cleaves the damaged DNA strand at the 3′ end [10]. The protein may also possess some functions in other cellular processes, including RNA polymerase II transcription and transcription-coupled DNA repair [11], [12]. Numerous studies indicate that a defective *ERCC5* plays a pivotal role in the initiation of carcinogenesis and that its deficiency leads to DNA repair defects, genomic instability, and failure of gene transcription modulation [13]–[15]. To date, some studies have reported that polymorphisms in *ERCC5* are associated with risk of cancers of the breast [16]–[18], lung [19]–[21], skin [22]–[24] and bladder [25], [26], but there is only one published study of a nonsynonymous SNP (rs17655) and esophagus cancer in Caucasians with a small sample size [27]. Furthermore, several studies suggested that variations in ERCC5 transcript and protein levels as well as *ERCC5* variant genotypes were associated with risk of squamous cell carcinoma of the head and neck (SCCHN) [28]–[30], which shares similar risk factors with ESCC. Therefore, we hypothesized that potential functional *ERCC5* SNPs are associated with ESCC risk, and we tested this hypothesis in an Eastern Chinese population.

## Materials and Methods

### Study Subjects

This hospital-based case-control study included 1115 cases of ESCC and 1117 cancer-free controls. All subjects were genetically unrelated ethnic Han Chinese from Eastern China, including Shanghai, Jiangsu province and the surrounding regions. Patients newly diagnosed with histopathologically confirmed primary ESCC were recruited from Fudan University Shanghai Cancer Center (Shanghai, China) between March 2009 and September 2011. Blood samples of all patients were collected and processed by the Institutional Tissue Bank at Shanghai Cancer Center as a daily routine practice. Patients who had primary tumors outside the esophagus, tumors of an unknown origin, or any histopathologic diagnosis other than ESCC were excluded. Cancer-free controls were all recruited from a large prospective cohort - the Taizhou Longitudinal Study (TZL) in Taizhou, Jiangsu province, China, during the same period for the recruitment of cases [31]. Control subjects were frequency matched to the cases by age (±5 years) and sex. The response rates for cases and controls were approximately 93.0 and 90.0%, respectively. Each participant was asked to sign a written informed consent or next of kin or guardians consented on the behalf of participants whose capacity to consent was reduced, before we interviewed each eligible participant to obtain data on demographic characteristics and environmental exposure history, such as age, sex, ethnicity, body mass index (BMI), smoking and alcohol consumption. Individual who smoked <100 cigarettes in their lifetime were considered “non-smokers”, and the others were considered “smokers”. Similarly, subjects who drank alcoholic beverages at least once a week for ≥1 year were defined as “drinkers”, and the others were “non-drinkers”. Additionally, BMI was divided into two groups of “<24.0 and ≥24.0 kg/m^2^” according to the recommendations of the Working Group on Obesity in China, in which the BMI cut-off point for Chinese is 24 kg/m^2^ for overweight [32]. At the end of the interview, a 10-mL venous blood sample was collected from each subject. The research protocol was approved by the Institutional Review Board of the Shanghai Cancer Center, Fudan University.

**Table 1:** Distributions of selected variables in ESCC cases and cancer-free controls.

Variables	Cases No. (%)	Controls No. (%)	*P* a
All subjects	1115 (100)	1117 (100)	
Age, yr (Mean ± SD)^b^	60.40±8.31	60.76±10.26	0.110
≤50	137 (12.28)	155 (13.88)	
>50–≤60	417 (37.40)	402 (35.99)	
>60–≤70	425 (38.12)	392 (35.09)	
>70	136 (12.20)	168 (15.04)	
Sex			0.100
Males	901 (80.81)	871 (77.98)	
Females	214 (19.19)	246 (22.02)	
Smoking status			0.0004
Yes	687 (61.61)	605 (54.16)	
No	428 (38.39)	512 (45.84)	
Drinking status			<0.0001
Yes	494 (44.30)	364 (32.59)	
No	621 (55.70)	753 (67.41)	
Pack-years			<0.0001
0	424 (38.03)	512 (45.84)	
≤16 (mean)	151 (13.54)	244 (21.84)	
>16 (mean)	540 (48.43)	361 (32.32)	
BMI			<0.0001
<24.0	633 (56.77)	433 (38.76)	
≥24.0	482 (43.23)	684 (61.24)	

ESCC = esophageal squamous cell carcinoma; SD = standard deviation; BMI = body mass index.

a Two-sided *χ^2^* test for distributions between cases and controls.

### SNP Selection

Because the published genome-wide association studies (GWASs) of ESCC included all tagging SNPs of DNA repair genes but did not report any of such SNPs to be positively associated with cancer risk, our strategy in the present study was to include functional SNPs of *ERCC5*, a candidate gene rarely investigated for ESCC, that were not included in previously published GWASs. We searched the NCBI dbSNP database (http://www.ncbi.nlm.nih.gov/) and SNPinfo (http://snpinfo.niehs.nih.gov/) for putatively functional SNPs of *ERCC5* that are located in the regulatory region (i.e., the 5′ near gene, 3′ near gene, 5′ untranslated regions (UTR) and 3′ UTR) with a minor allele frequency (MAF) ≥5% in Chinese Han, Beijing (CHB) descendants. As a result, five SNPs (rs2094258, rs2016073, rs751402, rs2296147, and rs873601) were selected as previously described [33]. Although both rs2094258 and rs2016073 are predicted to have similar functions, rs2094258 has a higher allele frequency for CHB; therefore, we genotyped rs2094258 only but not rs2016073, although it is possible that rs2016073 may have some functionality that may result in different outcome in a larger association study. As a result, we genotyped three potentially functional SNPs (rs2094258C>T in the 5′ near gene, rs2296147T>C in the 5′ UTR, and rs873601G>A in the 3′ UTR), of which the two 5′ UTR SNPs were predicted to affect the transcription factor binding sites (TFBS) activity, and the 3′ UTR SNP may have an impact on splicing and miRNA binding sites. These three selected SNPs also captured other 20 untyped SNPs in the nearby genes [33], including one non-synonymous SNP (rs17655) that is the only one common SNP with MAF >5% in CHB (LD for rs873601 and rs17655; r^2^ = 0.910).

**Table 2:** Logistic regression analysis of associations between the *ERCC5* variant genotypes and ESCC risk.

Variants	Genotypes/Alleles	Cases	Controls	*P* a	Crude OR	*P*	Adjusted OR	*P* b
		(N = 1,115)	(N = 1,117)		(95% CI)		(95% CI)^b^	
rs2296147	TT	757 (67.89)	699 (62.58)	**0.016** c	1.00		1.00	
	CT	305 (27.35)	368 (32.95)		**0.77 (0.64–0.92)**	**0.004**	**0.76 (0.63–0.93)**	**0.006**
	CC	53 (4.75)	50 (4.48)		0.98 (0.66–1.46)	0.916	1.08 (0.71–1.63)	0.734
	CT/CC	358 (32.11)	418 (37.42)	**0.008** d	**0.79 (0.66–0.94)**	**0.008**	**0.80 (0.67–0.96)**	**0.016**
	T	1819 (0.82)	1766 (0.79)		1.00		1.00	
	C	411 (0.18)	468 (0.21)	**0.025**	**0.85 (0.73–0.98)**	**0.026**	**0.87 (0.74–1.01)**	**0.066**
rs2094258	CC	414 (37.13)	424 (37.96)	0.838^c^	1.00		1.00	
	CT	524 (47.00)	525 (47.00)		1.02 (0.85–1.23)	0.813	1.04 (0.86–1.25)	0.722
	TT	177 (15.87)	168 (15.04)		1.08 (0.84–1.39)	0.552	1.03 (0.79–1.34)	0.816
	TT/CT	701 (62.87)	693 (62.04)	0.686^d^	1.04 (0.87–1.23)	0.686	1.03 (0.87–1.24)	0.713
	C	1352 (0.61)	1373 (0.62)		1.00		1.00	
	T	878 (0.39)	861 (0.38)	0.602	1.03 (0.92–1.16)	0.602	1.02 (0.90–1.16)	0.771
rs873601	GG	314 (28.16)	311 (27.84)	0.956^c^	1.00		1.00	
	AG	566 (50.76)	565 (50.58)		0.99 (0.82–1.21)	0.937	1.00 (0.82–1.23)	0.988
	AA	235 (21.08)	241 (21.58)		0.97 (0.76–1.23)	0.775	0.95 (0.74–1.21)	0.664
	AG/AA	801 (71.84)	806 (72.16)	0.867^d^	0.98 (0.82–1.18)	0.867	0.99 (0.81–1.20)	0.876
	G	1194 (0.54)	1187 (0.53)		1.00		1.00	
	A	1036 (0.46)	1047 (0.47)	0.812	0.98 (0.87–1.10)	0.740	0.97 (0.86–1.10)	0.670

a Chi-square test for genotype distributions between cases and controls.

b Adjusted for age, sex, body mass index (BMI), smoking status, and drinking status in logistic regress models.

c for additive genetic models.

d for dominant genetic models.

### Genotyping

Genomic DNA was extracted from the buffy-coat fraction of the blood samples using the Qiagen Blood DNA Mini Kit (Qiagen Inc., Valencia, CA). All the three SNPs were genotyped using the Taqman real-time PCR method with a 7900 HT sequence detector system (Applied Biosystems, Foster City, CA). To ensure high genotyping accuracy, strict quality control procedures were implemented, and four duplicated positive controls and four negative controls (no DNA) were used in each of 384-well plates. Approximately 5% of the samples were repeatedly genotyped, and the results were 100% concordant.

### Statistical Methods

Differences in frequency distributions of the selected demographic variables, risk factors and each of alleles and genotypes of the selected SNPs between patients with ESCC and control subjects were evaluated using the χ^2^ test. Univariate and multivariate logistic regression analyses were used to calculate crude and adjusted odds ratios (ORs) to estimate the risk of ESCC and 95% confidence intervals (95% CIs). Multivariate adjustments were made, where appropriate, for age, sex, BMI, and smoking and drinking status, in further stratification analysis of genotype data. The heterogeneity of associations between subgroups was estimated using the Chi-square-based Q test. The Hardy–Weinberg equilibrium for genotype distribution in controls was tested by a goodness-of-fit χ^2^ test. Haplotype frequencies and individual haplotypes were estimated using unphased genotype data. The haplotype of the highest frequency was used as the reference group to calculate ORs for haplotype associated with ESCC risk in logistic regression analysis.

We also calculated the false-positive report probability (FPRP) to detect the false-positive association findings. For all the significant results, a prior probability of 0.01 was assigned to detect an OR of 1.56 (for a risk effect) or 0.67 (for a protective effect) for an association with genotypes and alleles of each SNP. Only significant results with an FPRP value less than 0.20 were considered a noteworthy association. Statistical significance was established at a *P*<0.05, and all tests were two-sided and performed with the SAS software (version 9.1; SAS Institute, Cary, NC).

## Results

### Population Characteristics

The final analysis included 1115 cases of confirmed ESCC and 1117 healthy controls, whose characteristics are summarized in **Table 1**. By the study design, there were no statistical differences in the distributions of age and sex between the cases (mean age of 60.40±8.31 years with 80.81% males) and controls (mean age of 60.76±10.26 with 77.98% males), indicating the frequency matching on age and sex was adequate (*P* = 0.110 and 0.100, respectively). However, the cases were more likely to be smokers (61.61% vs. 54.16%, *P* = 0.0004) and drinkers (44.30% vs. 32.59%, *P*<0.0001) than the controls. In addition, the cases exhibited a lower proportion of BMI ≥24.0 than the controls (43.23% vs. 61.24%, *P*<0.0001). Therefore, these variables were further adjusted for in subsequent multivariate logistic regression analyses.

### *ERCC5* Allele and Genotype Distributions and Association with ESCC Risk

The allele and genotype frequencies of the three selected SNPs in cases and controls are summarized in **Table 2**. All the observed genotype frequencies agreed with the Hardy–Weinberg equilibrium in the controls (*P* = 0.860 for rs2296147, *P* = 0.793 for rs2094258, and *P* = 0.601 for rs873601). The rs2296147 C allele and rs873601 A allele were less frequent among cases than among controls (0.18 vs. 0.21, *P = *0.025 and 0.46 vs. 0.47, *P = *0.812, respectively), whereas the rs2094258 T allele was more frequent among cases than among controls, though the difference was not statistically significant (0.39 vs. 0.38, *P = *0.602). In the single-locus analyses, only genotype frequency of rs2296147 was significantly different between the cases and the controls (*P* = 0.016). After adjustment for age, sex, BMI and smoking, and drinking status in multivariate logistic regression analysis, rs2296147 variant C genotypes were associated with a significantly decreased risk of ESCC (adjusted OR = 0.76, 95% CI = 0.63–0.93, *P* = 0.006 for CT vs. TT and adjusted OR = 0.80; 95% CI = 0.67–0.96, *P* = 0.016 for CT/CC vs.TT). In addition, the rs2296147 variant C allele was borderline significantly associated with a lower risk of ESCC (adjusted OR = 0.87, 95% CI = 0.74–1.01, *P* = 0.066). However, there was no evidence of an association between other two SNPs (rs2094258 and rs873601) and overall ESCC risk.

### Stratification Analysis of ESCC Risk by *ERCC5* Polymorphisms

We further evaluated the association between *ERCC* SNPs and ESCC risk stratified by selected variables including age, sex, BMI, smoking status and drinking status. As shown in **Table 3**, the stratification analysis indicated that the protective effect of rs2296147 CT/CC genotypes was more evident in older subjects (adjusted OR = 0.75, 95% CI = 0.57–0.97), and never-drinkers (adjusted OR = 0.75, 95% CI = 0.59–0.95), BMI <24.0 (adjusted OR = 0.77, 95% CI = 0.59–1.00), compared with the TT genotype. However, further homogeneity tests did not show statistical evidence for any differences (*P*>0.05) between every two strata, suggesting no risk modification by the variables under investigation. Additionally, no associations were observed between the genotypes of other two SNPs (rs2094258 and rs873601) and risk of ESCC in the stratification analysis, nor was there any statistical evidence for gene-environment interactions between the variant genotypes and these variables on risk of ESCC (**data not shown**).

**Table 3:** Stratification analysis for associations between *ERCC5* variant genotypes and ESCC risk.

Variables	rs2296147 (cases/controls)		Adjusted OR^a^ (95% CI)	*P* a	rs2094258 (cases/controls)		Adjusted OR^a^ (95% CI)	*P* a	rs873601 (cases/controls)		Adjusted OR^a^ (95% CI)	*P* a
	TT	CT/CC			CC	TT/CT			GG	AG/AA		
Age, yr												
≤60 (median)	361/344	193/213	0.87 (0.67–1.13)	0.31	211/221	343/336	1.06 (0.82–1.37)	0.71	149/147	405/410	1.06 (0.80–1.41)	0.92
>60 (median)	396/355	165/205	0.75 (0.57–0.97)		203/203	358/357	0.98 (0.76–1.26)		165/164	396/396	0.95 (0.72–1.24)	
Sex												
Males	608/545	293/326	0.83 (0.68–1.02)	0.64	338/336	563/535	1.04 (0.85–1.28)	0.88	252/243	649/628	1.00 (0.81–1.25)	0.79
Females	149/154	65/92	0.66 (0.43–1.01)		76/88	138/158	1.13 (0.75–1.72)		62/68	152/178	0.93 (0.60–1.44)	
Smoking status												
0	287/319	137/193	0.81 (0.61–1.07)	0.95	161/193	263/319	1.02 (0.77–1.34)	0.76	114/136	310/376	0.96 (0.71–1.30)	0.87
≤16 (mean)	110/148	41/96	0.59 (0.37–0.94)		56/98	95/146	1.09 (0.71–1.69)		48/66	103/178	0.77 (0.49–1.23)	
>16 (mean)	360/232	180/129	0.84 (0.62–1.14)		197/133	343/228	1.05 (0.78–1.41)		152/109	388/252	1.15 (0.83–1.58)	
Drinking status												
Never	429/469	192/284	0.75 (0.59–0.95)	0.38	225/288	396/465	1.10 (0.88–1.38)	0.49	176/197	445/556	0.88 (0.69–1.13)	0.16
Ever	328/230	166/134	0.83 (0.61–1.13)		189/136	305/228	0.97 (0.72–1.31)		138/114	356/250	1.18 (0.86–1.61)	
BMI												
<24.0	434/273	199/160	0.77 (0.59–1.00)	0.80	226/161	407/272	1.07 (0.83–1.39)	0.61	172/120	461/313	1.04 (0.79–1.38)	0.51
≥24.0	323/426	159/258	0.82 (0.64–1.06)		188/263	294/421	0.97 (0.76–1.24)		142/191	340/493	0.94 (0.73–1.23)	

a Obtained in logistic regression models with adjustment for age, sex, BMI, smoking status and drinking status.

b *P* for heterogeneity test using the Chi-square-based Q test.

### *ERCC5* Haplotypes and ESCC Risk

We found that the LD among these three SNPs of *ERCC5* was incomplete in all control subjects (rs2094258 and rs2296147: r^2^ = 0.163; rs2094258 and rs873601: r^2^ = 0.438; rs873601 and rs2296147: r^2^ = 0.102), suggesting that variant haplotypes may play a role in cancer risk. We inferred eight *ERCC5* haplotypes, four of which had a frequency less than 0.05 and were merged into one group of “minor haplotypes” for further analysis. However, no *ERCC5* haplotype in this study population was associated with ESCC risk, when the most common haplotype T-T-G was used as the reference (**Table 4**).

Finally, the FPRP values were calculated at different prior probability levels for all significant findings. For a prior probability of 0.01, assuming the OR for specific genotype or allele was 0.67, with statistical power of 0.918, 0.963 and 0.998, the FPRP values were 0.301, 0.451 and 0.721, respectively, for an association of the CT, CT/CC genotypes and C allele with a reduced risk of ESCC in all individuals (**Table 5**).

**Table 4:** Haplotype analysis for genotypes of *ERCC5* and ESCC risk.

Haplotype	Haplotype frequencies				Crude OR (95% CI)	*P*	Adjusted OR (95% CI)^a^	*P* a
	Cases (N = 2230)		Controls (N = 2234)					
	N	%	N	%				
T-T-G	835	37.44	824	36.88	1.00		1.00	
C-C-A	361	16.19	397	17.77	0.90 (0.76–1.07)	0.217	0.90 (0.75–1.08)	0.263
T-C-A	634	28.43	613	27.44	1.02 (0.88–1.18)	0.785	1.02 (0.88–1.19)	0.760
T-C-G	309	13.86	293	13.12	1.04 (0.86–1.25)	0.675	1.06 (0.87–1.29)	0.577
All minor haplotypes^b^	91	4.08	107	4.78	0.84 (0.63–1.13)	0.246	0.88 (0.65–1.20)	0.414

a Obtained in logistic regression models with adjustment for age, sex, BMI, smoking status and drinking status.

b Including four haplotypes, each with a frequency <0.05.

**Table 5:** False-Positive Report Probability Values for Associations between ESCC risk and the Frequency of Genotypes and Allele of *ERCC5* rs2296147.

Genotype/Allele	OR^a^ (95% CI)	*P* value^b^	Statistical Power^c^	Prior probability				
				0.2500	0.1000	0.0100	0.0010	0.0001
*ERCC5* rs2296147								
CT versus TT	0.77 (0.64–0.92)	0.004	0.918	0.013	0.038	0.301	0.813	0.978
CT/CC versus TT	0.79 (0.66–0.94)	0.008	0.963	0.024	0.070	0.451	0.892	0.988
C versus T	0.85 (0.73–0.98)	0.026	0.998	0.072	0.190	0.721	0.963	0.996

a The crude OR reported in Table 2.

b The omnibus chi-square test of the genotype and allele frequency distributions reported in Table 2.

c Statistical power was calculated using the number of observations in the study and the OR and *P* values in this table.

## Discussion

In the present case-control study, we investigated the associations between three common, putatively functional SNPs of the *ERCC5* gene and the risk of ESCC in an Eastern Chinese population. When we evaluated each SNP separately, only the rs2296147 variant CT/CC genotypes were associated with significantly reduced ESCC risk, but this risk was not observed for the other two rs2094258C>T and rs873601G>A SNPs. To the best of our knowledge, this is the first study that investigated the association of these three *ERCC5* SNPs with the ESCC risk.

Given the role of the *ERCC5* gene in the NER pathway, it is biologically plausible that functional *ERCC5* SNPs may contribute to ESCC susceptibility. To date, only three reported studies have investigated the association between rs2296147 and risk of cancers other than ESCC, of which one was conducted for SCCHN with 1059 white patients and 1066 healthy controls [30]; another included mixed populations with 722 cases of endometrial cancer and 726 cancer-free controls [34]; and the third was performed in a Chinese population with 1125 gastric cancer cases and 1196 healthy controls [33]. Although the rs2296147 C allele was associated with a non-significantly altered risk of cancers in these three reported studies of other cancers, the rs873601 A variant genotypes were found to be associated with an increased risk of gastric cancer [33]. For the rs2094258C>T SNP, only one study investigated its association with risk of SCCHN, but the result was null, which is in agreement with the finding in the present study of ESCC. The possible discrepancy of the results among different studies may be due to different genetic backgrounds and etiologies. For example, the frequency of the rs2296147 C allele in non-Hispanic White populations was 0.47 in the study by Ma et al. [30] but was 0.20 in the present study (in a Chinese population). Another possibility may be related to different etiologies of the primary tumors. Although some studies have shown that some sequence variants in the region of chromosome 5p15.33 and 8q24 are associated with risk of different cancer types [35]–[39], most published studies reported that the same SNP may be cancer-specific. It is likely that our present study still had a limited statistical power to detect a weak effect. For instance, for the sample size in the present study, we had only a 54.9% power to detect OR = 1.23 for rs873601 under a recessive model as reported by He et al. [33]. Meanwhile, greater FPRP values were observed for the significant associations between rs2296147 C variant genotypes and ESCC risk, which further suggests a relatively small sample size in the present study. Therefore, whether or not an association between *ERCC5* SNPs with cancer risk is cancer-specific needs to be validated in additional large studies of different cancer types, either in additional groups of homogenous ethnicity or in more ethnically diverse groups.

Moreover, in the published GWASs for cancers, including ESCC, based on common tagging SNPs, none of the SNPs in NER genes has been identified as susceptibility locus. This poses a challenge to the common variants and common diseases theory [40]. Because NER genes are well-known susceptibility genes, it is plausible that individuals carrying NER variants may be prone to exposures that instigate damage to DNA and thus to cancer. Consequently, without comprehensive information about such exposures for additional adjustment or stratification, the actual associations may be either biased or masked, as likely to be in the published GWASs. XP patients, for instance, can greatly decrease their risk of developing skin cancer, if they can effectively avoid sun exposure in their lifetime. Alternatively, common variants are unlikely to have a significant biological effect or when their effects are weak, sufficient study power is needed, even in the GWASs. For example, when 500 000 SNPs were analyzed in a GWAS, multiple comparisons demand a *P*-value less than 10^−7^ or 10^−8^ to avoid finding false significant associations, which markedly decreases the power to detect SNPs associated with the disease phenotype [41]. This is simply because common variants may moderately contribute to risk of cancer due to their weak penetrance. In that case, the identification of additional rare but functional variants requires a single large GWAS or combined analysis of several published GWASs in the future.

There is some biological plausibility that functional genetic variants, like those of *ERCC5*, may contribute to risk of carcinogenesis initiated by carcinogen-induced DNA damage. Under such circumstances, a candidate approach, rather than a GWAS, may be more appropriate and feasible. Acting as a structure-specific endonuclease and also a 5′-3′ exonuclease, the ERCC5 protein is required for two sub-pathways in NER. One is transcription-coupled DNA repair (TCR), which preferentially removes DNA damage in the transcribed DNA strand of active genes; the other is global genomic repair (GGR), which removes lesions throughout the genome [42], [43]. It has been observed that rare mutations in *ERCC5* are causative of DNA repair deficient diseases characteristic of abnormal apoptosis and a high level of cancer risk [11], [12]. Additionally, *ERCC5* has also been shown to play a role in the regulation of transcription [11], [44]–[46]. Numerous studies have reported that altered *ERCC5* transcript expression modulate transcription domain-associated repair capacity [13], [47] and other important phenotypic effects, including inter-individual variation in the incidence of several cancer types [28], [29].

Polymorphic site for rs2296147 is located in the 5′UTR of *ERCC5* and is predicted to be a putative P53 consensus site [48]. Although biological significance of this SNP has not been elucidated, a growing body of evidence has indicated that SNPs located at the TFBS are likely to affect gene expression by altering DNA binding properties of transcription factors and thus may contribute to susceptibility to cancer [49], [50]. Indeed, previous functional studies have revealed that cis-acting genetic variation at a putative TP53 recognition site (for the rs2296147 T allele) and that an E2F1/YY1 response site (for the rs751402 A allele) is associated with higher levels of allele-specific *ERCC5* transcripts in the normal human bronchial epithelium among lung cancer patients [51], which is in agreement with our current finding that the rs2296147 C allele may be protective against ESCC. Interestingly, expression data from the HapMap suggested a nonsignificant correlation of the rs2296147 C variant genotypes with low *ERCC5* mRNA expression levels in EBV-transformed lymphoblastoid cell lines [33]. The possible reason might be that the functional effect of rs2296147 is modest and may be in LD with either other untyped functional SNPs, thereby altering the function of *ERCC5*, or with an adjacent susceptibility gene. Another possibility may be that the study with a relatively small sample size has a limited statistical power to detect a weak effect. Additionally, rs2296147 in the 5′UTR may have some effects on regulating posttranscriptional processing, because the 5′UTR length of *ERCC5* may be critical for its recruitment to the polyribosomal cellular fraction that could increase translation in response to the protein kinase C activity as a result of DNA damage response [52]. However, it would be more conclusive, if we had data on ERCC5 mRNA/protein expression by genotypes. Unfortunately, the target tissues were not made available to us at the time the study initiated. Taken together, because we do not have directly biological evidence, it may be premature to conclude that rs2296147 is a causal SNP.

There are several potential limitations in the present study that should be addressed. First, although our study was relatively large, the small sample size in subgroup analysis may have limited statistical power to detect significant associations for each of the strata, let alone the power to assess gene-gene or gene-environment interactions adequately. Second, we used common, potentially functional SNPs instead of tagging SNPs, which may miss some important genetic variations within the gene. Third, because it was a hospital-based case-control study, there may be inherent biases for selection of the nonrepresentative population and retrospective collection of exposure data. Finally, because of our tissue access constraint, this study did not include the detection of the influence of the *ERCC*5 variants on its protein expression in the target tissue. These limitations can only be overcome in larger, well designed prospective studies in the future.

In summary, our findings suggested that *ERCC5* polymorphisms may contribute to risk of ESCC among Eastern Chinese populations, but the effect was weak and needs to be further validated in larger studies in the future.
